# Vital and Clinical Signs Gathered Within the First Minutes After a Motorcycle Accident on a Racetrack: an Observational Study

**DOI:** 10.1186/s40798-021-00350-6

**Published:** 2021-08-21

**Authors:** Karin Hugelius, Jerry Lidberg, Linda Ekh, Per Örtenwall

**Affiliations:** 1 Faculty of Medicine and Health, Örebro University, 70182 Örebro, SE Sweden; 2 Region Örebro County, Karlskoga Hospital, 691 44 Karlskoga, Sweden; 3 TM: s ambulance, 652 26 Karlstad, Sweden; 4 Department of Clinical Physiology, Region Jämtland Härjedalen, 831 31 Östersund, Sweden; 5 Sahlgrenska Academy, Gothenburg University, 405 30 Gothenburg, Sweden

**Keywords:** Trauma, Motorcycle accidents, Vital signs, Prehospital care, Ambulance, Trauma triage

## Abstract

**Background:**

Little is known about vital signs during the very first minutes after an accident. This study aimed to describe the vital signs of motorcycle riders shortly after racetrack crashes and examine the clinical value of these data for the prehospital clinical assessments.

**Methods:**

A retrospective observational cohort based on data from medical records on 104 motorcycle accidents at a racetrack in Sweden, covering the season of 2019 (May 01 until September 17), was conducted. Both race and practice runs were included. In addition, data from the Swedish Trauma Registry were used for patients referred to the hospital. Kruskal-Wallis test and linear regression were calculated in addition to descriptive statistics.

**Results:**

In all, 30 riders (29%) were considered injured. Sixteen riders (15%) were referred to the hospital, and of these, five patients (5% of all riders) had suffered serious injuries. Aside from a decreased level of consciousness, no single vital sign or kinematic component observed within the early minutes after a crash was a strong clinical indicator of the occurrence of injuries. However, weak links were found between highsider or collision crashes and the occurrence of injuries.

**Conclusion:**

Except for a decreased level of consciousness, this study indicates that the clinical value of early measured vital signs might be limited for the pre-hospital clinical assessment in the motorsport environment. Also, an adjustment of general trauma triage protocols might be considered for settings such as racetracks. Using the context with medical professionals at the victim’s side within a few minutes after an accident, that is common in motorsport, offers unique possibilities to increase our understanding of clinical signs and trauma in the early state after an accident.

## Key Points

- Except for a decreased level of consciousness, this study indicates that the clinical value of early measured vital signs to determine the severity of injuries after road racing crashes on racetracks might be limited.
- Type of crash seems to be more significant than speed as indicators for severe injuries on motorcycle crashes at racetracks.
- Study settings with medical professionals at the victim’s side within a few minutes after an accident offers unique possibilities to increase our understanding of clinical signs and trauma.

## Background

In 2016, 1.35 million persons globally were killed in road traffic deaths. Of these, about 28% were motorcycle riders [1]. In Sweden, 47 motorcyclists were killed and over 200 were injured during 2018 [2]. Road racing is a motorsport where motorcycles race on paved road surfaces, most often on a racetrack. However, little is known on the prevalence and outcome of accidents from road racing track accidents. One study on crashed in road racing grand prix indicates that 12–14% of all riders suffered a crash during racing [3]. In accordance with road racing regulations, medical teams are often present at racetracks to ensure an immediate emergency response in case of a crash [4]. In such situations, the first professional medical assessment can be made within a few minutes; this is opposed to motorcycle crashes on public roads, where the first public ambulance might arrive significantly later [5].

Prehospital assessment of trauma patients is a crucial part of the trauma care chain [6]. Most prehospital assessment protocols use a combination of vital signs, detection of certain anatomic injuries, injury mechanism and (sometimes) age [5]. However, little is known about vital and clinical signs in the very early stage after an accident, due to lack of studies and data. Kinetic energy is related to the square of the velocity of a moving object. On racing tracks, speeds may be in the range of 200 km/h, with riders having corresponding extreme energy levels. To address this, protection measures, including safety zones and air fences (inflatable air barriers that protect bikers from hitting a solid fence), are designed to gradually reduce speed and thus energy. Moreover, requirements for wearing personal protective gear, such as helmets, have been introduced to minimize serious injuries [7, 8]. These aspects might affect the clinical assessment of road racing riders. By describing and analysing vital and clinical signs within a few minutes of an accident, a greater understanding of their value for the prehospital clinical assessment in the early state after an accident can be obtained.

This study aimed to describe the vital and clinical signs of motorcycle riders shortly after racetrack crashes and examine the clinical value of these data for prehospital clinical assessments.

## Methods

A retrospective observational cohort study was conducted by analysing medical records from 104 motorcycle crashes on a Swedish racetrack during the road racing season of 2019.

### Study Setting

The racetrack used in this study was a permanent racetrack in Sweden. The medical teams consisted of nurses and physicians with experience in prehospital trauma care. In order to drive a motorcycle at the racing track, personal protective gear, such as a helmet and back protectors, was required.

### Participants

Data from all motorcycle crashes on the racetrack during the season of 2019 (May 01 until September 17)—when a medical team was present, and therefore, a medical record existed—were included. Crashes from races (national championships and regional/ local competitions) as well as practice runs were included. No private or commercial testing activities occurred on the actual racetrack during the season.

### Data Collection Procedure

Data were collected retrospectively from paper medical records taken at the racetrack. Information on vital signs, clinical injury signs, type of crash and speed at crash was collected from the paper records and imported to the statistical programme SPSS (IBM Corp., released 2017. IBM SPSS Statistics for Windows, Version 25.0. Armonk, NY: IBM Corp). Three types of crashes [7] were studied: lowsider crashes, or crashes resulting from a loss of traction, where the motorcycle went down on the side closer to the ground; highsider crashes, involving sudden and violent long bike axis rotations, where the bike was oversteered into a turnover; and collisions with other bikers. If the riders were unable to specify actual speeds for their crashes, an estimation based on the specific location around the racetrack was used. For this purpose, time-tracking systems and helmet cameras providing speed measurements from ten professional and semi-professional motorcycle riders were used to determine the mean speed at all locations around the racetrack.

All vital signs were measured by members of the medical team. Clinical signs of injury were defined as documented pain or observed obvious injuries, such as paradoxical breathing, extremity deformities or external bleeding. For patients referred to an emergency hospital, information on their condition (measured with injury severity score (ISS) [9] and new injury severity score (NISS) [9]), documented type of injuries and status at discharge from hospital was collected from the Swedish Trauma Registry (SweTrau) and integrated into the SPSS dataset.

In order to ensure that all serious accidents at the racetrack during the observed season were included, the Nordic Motorsport Council and the insurance company that covers licensed racing motorcyclists were contacted and asked if they had any knowledge of reported injuries or fatalities of motorcycle drivers due to crashes at that specific racetrack aside from those already included. In addition, public ambulance alarm records for the racetrack were also scanned for accidents not included in the current data.

### Analysis

For descriptive analysis, percent proportions and, when applicable, means, ranges, standard deviations (SD) and a 95% confidence interval (CI) were used to describe participant demographics, injuries and other information. Due to a small study sample and outliers, the Kruskal-Wallis test was used to analyse any differences between vital sign means among uninjured, injured and severely injured riders. Bonferroni post hoc analysis was used to determine significant differences between groups. Multiple regression analyses were conducted to calculate the association of speed at crash, type of crash, age of the rider, occurrence of clinical signs of injury and abnormal first vital signs (GCS, RR, Sp02, HR and SBP) measured within the first 10 min from the crash (*n*=62) with the occurrence of injuries (dependent variable). The exact analysis used and outcome variables are presented in each table or in the results. Injuries were defined as any signs of injury documented in the medical records or recorded, based on ISS, in the SweTrau. Mild injuries were defined as those with an ISS of 1–14, and severe injuries were defined as those with an ISS > 15 [7]. A confidence interval of 95% and a statistical significance of *p* ≤ 0.005 were used with regard to the limited observations included [10]. No post hoc test was conducted, but the statistical analysis was verified by an external statistician.

Ethical approval was obtained from the Swedish Ethical Review Authority (dnr 2019-05803).

## Results

Data from 104 motorcycle crashes were analysed. The gender distribution was 97 (93%) males and 7 (7%) females with ages ranging between 14 and 64 years (mean 38 years, SD 14). Of the crashes, 42 (40%) occurred during racing and 62 (60%) occurred during practice runs. Lowsider crashes represented 67 (68%) accidents, highsider crashes accounted for 20 (20%) accidents and 12 (12%) were caused by collisions with other motorcycles (data were missing for five crashes). Crash speeds (actual speed stated by the rider *n*= 80, or estimated speed *n*=24) varied from 50 km/h to over 180 km/h, with a mean of 92 km/h (SD 24).

In all, 30 riders (29%) were considered injured. Of these, 25 patients (24%) had suffered injuries (ISS 1–14) and 5 (5%) sustained major trauma (ISS > 15). Sixteen riders were referred to the hospital as trauma patients. The mean ISS for those 16 patients was 8 (range 1–21; SD 6) and the mean NISS was 10 (range 1–29; SD 8.1). Five riders (5%) were classified as having major trauma (ISS > 15) (see Table 1). None were reported dead after 30 days. Neither the motorcycle or insurance organizations nor the public local ambulance services had knowledge of any accidents on the racetrack that were not already part of the data.

**Table 1:** Overview of outcomes for all riders

Outcome, *N*=104	*n* (%)
Considered injured	30 (29)
Transported from track to racetrack medical centre with ambulance*	35 (34)
Attended the racetrack medical centre by feet*	69 (66)
Cleared on the track and returned to paddock or race	79 (76)
Referred to hospital as trauma patients	16 (15)
Advised to seek to non-emergency medical care	9 (9)
Reported dead after 30 days	0 (0)

*In accordance with regulations, all riders who crashed had to visit the medical centre after the crash in order to get medical clearance

**Fig. 1:**
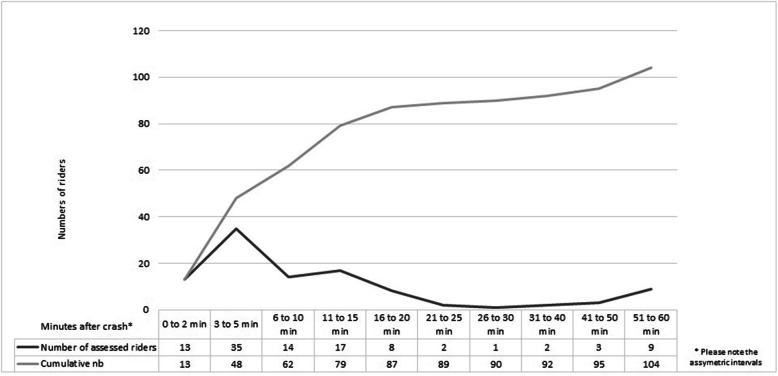
Overview of time for first documented vital signs

The first documented vital signs were reported between 2 and 58 min after crashes, with a mean of 15 min (SD 18). In 35 (34%) of the accidents, vital signs were reported within 3 min of the crash. After 5 min, 47 accidents (45%) had vital signs measured, and 78 cases (75%) were accounted for within 10 min of the crash. Just nine accidents (8%) had initial vital signs measured after 60 min (see Fig. 1).

### Vital Signs Within the First Minutes After an Accident

**Fig. 2:**
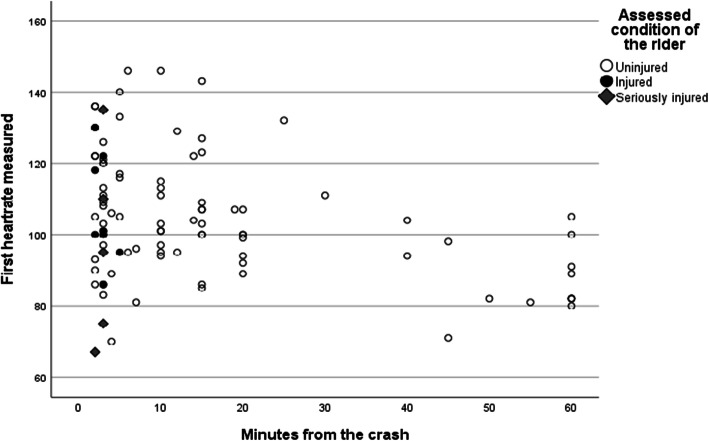
First measured heart rates and times after accidents

The vital signs observed within the first minutes after a crash varied considerably. The first measured heart rates for all riders and the times for those observations are displayed in Fig. 1. As shown, heart rates varied between 67 and 146 beats per minute (mean 105; SD 18). When comparing the first measured heart rates among seriously injured (marked in Fig. 2) and uninjured riders, no significant difference could be observed (Fisher’s exact test; uninjured riders: *n* = 67; M = 104; SD = 18; injured riders: *n* = 28; M = 105; SD = 18; 95% CI −7 to 9, *p* = 0.92).

The means of vital signs among uninjured, injured and seriously injured riders were compared using the Kruskal-Wallis test (see Table 2). A significantly lower Glasgow Coma Scale (GCS) was observed when comparing both uninjured (15; SD 0) with injured riders (12; SD 4) (Kruskal-Wallis test: H (2) = 19; *p* = 0.00; post hoc Bonferroni adjusted; *p* ≤ 0.00) and uninjured (15; SD 0) with seriously injured riders (13; SD 2) (Kruskal-Wallis test: H (2) = 19; *p* = 0.00; post hoc Bonferroni adjusted; *p* ≤ 0.00). An increased respiratory rate among seriously injured (mean 25; SD 6) compared to uninjured (mean 18; SD 2) riders was also observed (Kruskal-Wallis test: H (2) = 7.6; *p* = 0.22; post hoc Bonferroni adjusted; *p* = 0.02). No other vital signs were significantly different between the groups.

**Table 2:** Vital signs measured within the first 5 min among uninjured, injured and seriously injured riders

Vital sign	*n*	Uninjured Mean (range, SD) (95% CI)	Injured Mean (range, SD) (95% CI)	Seriously injured Mean (range, SD) (95% CI)	*p*-value*
**Heart rate**	44	109 (70–140, SD 17) (95% CI 102–116)	107 (86–130, SD 16) (95% CI 92–102)	102 (67–135, SD 28) (95% CI 56–147)	0.34
**Respiratory rate**	40	18 (16–22, SD 2) (95% CI 17–18)	18 (16–22, SD 2) (95% CI 16–21)	25 (18–32, SD 6) (95% CI 18–25)	0.02**
**SpO2**	46	96 (94–98, SD 1) (95% CI 95–96)	95 (92–98, SD 2) (95% CI 93–97)	94 (90–97, SD 3) (95% CI 90–99)	0.82
**Systolic blood pressure**	43	139 (104–189, SD 20) (95% CI 131–147)	141 (105–190, SD 32) (95% CI 112–171)	133 (112–163, SD 23) (95% CI 96–170	0.08
**GCS**	48	15 (15–15, SD 0) (95% CI 15–15)	12 (3–15, SD 4) (95% CI 11–15)	13 (12–15, SD 2) (95% CI 11–16)	< 0.00**

*SpO2*, peripheral capillary oxygen saturation; *SBT*, systolic blood pressure; *GCS* Glasgow Coma Scale

*Analysed with the Kruskal-Wallis test. **For significant results, post hoc tests using Bonferroni were made to compare pairs within the groups (see text)

The development of vital signs for seriously injured patients during the first 15 min after an accident is shown in Table 3. Despite abnormal signs (as defined by the Swedish Trauma Protocol) in the very early phases, most vital signs were normalized when patients arrived at the hospital (see Table 3).

**Table 3:**
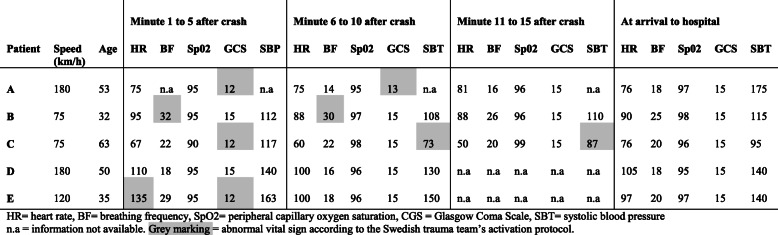
Overview of seriously injured riders’ vital signs during the first 15 min after a crash

*HR*, heart rate; *BF*, breathing frequency; *SpO2*, peripheral capillary oxygen saturation; *GCS*, Glasgow Coma Scale; *SBT*, systolic blood pressure; *n.a*, information not available; grey marking, abnormal vital sign according to the Swedish trauma team’s activation protocol

### Prehospital Clinical Signs of Injuries

Of all 104 riders who crashed, 74 (71%) had no signs of injury at all, while 30 (29%) were considered as injured. The injury location was six (20 %) suffering from head injury, seven (23 %) with upper extremity injuries (i.e. arm, hand or shoulder), 10 (33 %) with lower extremity damage (i.e. leg or foot), five (17 %) with thoracic injuries and two (6 %) suffering from suspected spinal cord injury. When comparing prehospital and hospital assessments of injury locations, there was a 75% match for the 16 patients transferred to the hospital. Five of the patients (31%) had additional injuries identified by the hospital trauma team as compared to the documented prehospital assessment. These included fractures or soft tissue injured on more locations than identified prehospital. None of the additional injuries found were considered as life threatening or severe on their own but added severity to the overall condition of the injured rider.

### Association Between Trauma Mechanisms, Vital Signs Measured Early and Injuries

The occurrence of injuries was associated with highsider or collision crashes, a prehospital decreased level of consciousness (GCS **<** 15) and clinical signs of injuries; the type of crash had the greatest impact (see Table 4).

**Table 4:** Correlation between first measured vital signs within 10 min from the crash, speed, age of rider, type of crash and occurrence of injuries

	***B***	95% CI	*t*	Sig.
**Speed ≥ 90 km/h**	0.02	(−0.06; 0.06)	0.56	0.59
**Highsider or collision**	0.92	(0.00; 0.19)	1.94	*0.05*
**Age ≥ 40 years**	−0.12	(−0.11; 0.85)	−0.25	0.81
**GCS < 15**	0.27	(0.74; 0.46)	2.75	*0.00*
**Respiratory rate** **< 10 or > 29**	0.15	(−0.14; 0.44)	1.04	0.30
**SpO2 < 95%**	0.03	(−0.18; 0.25)	0.31	0.76
**Heart rate** **< 50 or > 120**	−0.10	(−0.20; −0.00)	−2.24	0.08
**SBT< 90**	0.13	(−0.03; 0.29)	1.56	0.17
**Clinical signs of injury**	0.68	(0.57; 0.78)	12.6	*0.00*

Model summary: *p* = 0.00, *R*^2^ = 0.93. Outcome variable: injuries

*B*, unstandardized coefficient beta; *CI*, confidence interval (lower bound; upper bound); *Sig.*, *p* value; cursive number, significant *p* value. *GCS*, Glasgow Coma Scale; *SBT*, systolic blood pressure; *SpO2*, peripheral capillary oxygen saturation

### Dropouts

A few patients had incomplete vital sign documentation in any or several readings (heart rate (*n*=3), breathing frequency (*n*=2), systolic blood pressure (*n*=3)) and three (*n*=3) had no documented SpO2 assessments but all other data. No specific dropout analysis was conducted due to the small number of participants.

## Discussion

This study has shown that about 30% of all road racing crashes on the racetrack during practice and racing caused injuries, and in 5% of these, the riders were severely injured. Vital signs in the early phases after crashes varied significantly. Aside from a decreased level of consciousness, no single vital sign or kinematic component observed shortly after a crash was a strong indicator of neither injuries nor severe injuries. However, weak links may exist between highsider or collision crashes and the occurrence of injuries.

A decreased level of consciousness was more common among injured or severely injured riders compared with uninjured riders and was associated with the occurrence of injuries in the regression analysis. The results showed that the injured group had a lower mean GCS that the serious injured group. Also, it should be noted that two riders had normal GCS both at the racetrack and when arriving to hospital but were still severely injured. None of these two was diagnosed with traumatic brain injury. Since the number of injured among these subgroups were small, no conclusions can be drawn from these differences. The GCS has been found to show strong agreement when prehospital assessments were compared with emergency department assessments [11]. However, the patients in this study who presented a prehospital lowered level of consciousness showed normal levels when arriving at the hospital. It should be noted that in this study, medical professionals reached the patients within minutes of their crashes and could make professional assessments of consciousness levels. Normally, when ambulances arrive awhile after accidents, assessments of early decreased consciousness must rely on information gathered from bystanders or patients themselves. Such information may be less reliable or unavailable, depending on the circumstances. The current findings might be due to the presence of medical professionals, or from the very early clinical assessment made. The use of vital signs in prehospital assessments of trauma patients has been questioned [12, 13]. Within a few minutes of high-speed motorcycle crashes, vital sign variation was considerable. Some uninjured riders had heart rates over 120 during the first minutes without being seriously injured, while others had normal vital signs but were severely injured. One reason for this finding may be the physical demands of road racing, which make it difficult to distinguish between pathological high heart or respiratory rates shortly after a crash, or the physical fitness of the riders. Vital signs among the severely injured riders showed some abnormal values but also considerable variation during the first 15 min. Since clinical decision-making in the prehospital context is a complex process [14] where each patient must be examined thoroughly to avoid any misinterpretation of conditions [6, 14] repeated observations and trends over time may be more reliable and of greater clinical value than single measurements for predicting injury severity in a very early phase. This study could not confirm if vital signs measured later were more reliable as indicators of injury severity than vital signs measured early. Neither the observed mismatch of injury location nor the clinical effects of this mismatch could be concluded from the available data. However, the results indicate that timing and context must also be considered in such assessments. This is important to consider for medical teams on standby in high-risk sports.

According to the Swedish trauma team’s activation protocol, crash speeds over 35 km/h require trauma team activation for motorcycle accidents [15]. In this study, crashes occurred at actual or estimated speeds from 50 to 180 km/h, but no association was found between speed and the occurrence of injuries. However, 23% of the speed data in this study relied on estimated speeds. The estimations were based on professional or semi-professional riders and might therefore not be representative for nonprofessional riders. Despite these limitations, the findings indicate that crash speeds may not be a good indicator to previse the severity of motorcycle injury on racetracks. By contrast, the results suggest that highsider and collision crashes could be a better indicator in these settings; this was also observed by Bedolla et al. [6]. An adjustment of general trauma triage protocols could therefore be considered for specific contexts such as racetrack accidents, in order to provide guidance on prehospital trauma triage.

There are significant differences between racetracks and public roads. At racetracks, bikers are often experienced and trained, having safety zones and mandatory personal protection gear that gradually dissipates energy in case of a crash. Motorbikes on public roads must share the road with other types of vehicles, some travelling in the opposite direction. Moreover, public bikers have the choice of going without protective gear. Another difference is that professional or semi-professional riders most often are elite athletics. Therefore, generalizing these findings to public motorcycling should be done with caution. The low rate of serious injuries despite extremely high speeds could indicate that the protective measures on racetracks are effective in reducing the risk of severe injuries compared with public roads. Regardless, crashes at racetracks offer a special opportunity to collect early crash data and may provide valuable insights into motorcycle crashes and prehospital clinical assessments that could benefit the larger, non-competitive motorcycle riding population [6, 16]. The study’s setting, where medical professionals were able to respond within minutes of an accident, offers knowledge that cannot be obtained easily in ordinary prehospital trauma care or registers. Similar settings are available in other sports, such as alpine skiing. Using data from these settings offers a unique possibility to further understand the initial timeframe after accidents. Therefore, this study has value despite the limited number of crashes included. The results of this study raise several questions concerning prehospital clinical assessment of trauma patients. Further research should be undertaken to investigate the development over time of vital signs in trauma patients and further discuss their value for prehospital assessment. Additionally, the value of kinematic data in prehospital trauma triage protocols for different settings and activities needs to be further explored.

### Limitations

The study has several limitations. First, the number of severely injured patients in this study sample was very small, reducing the possibility of generalizing the results. However, this is also a finding itself, that fewer serious injuries than expected occurred at the racetrack. The available data only cover accidents that occurred on a racetrack when medical professionals were on standby. Despite regulations, it cannot be excluded that any road racing activities were conducted without the medical team on standby, and therefore not covered in this study. In addition, there may have been late complications or injuries requiring medical care that were not covered in this study—for example, if the patient had sought medical care in another country (e.g. their home country). However, the screening of insurance companies, motorcycle organizations and public ambulance services showed that they had no information on fatalities due to accidents on racetracks during the period studied. The SweTrau register reports both ISS and NISS for each incident. As the ISS and NISS have been reported to have a similar ability of predicting mortality in trauma patients [17], this study used the ISS only to define severe injuries in the analysis.

## Conclusions

Vital signs measured within a few minutes of riders being involved in motorcycle crashes on a racetrack varied considerably. Except for a decreased level of consciousness, the clinical value for prehospital assessment of vital or clinical signs measured early might, therefore, be limited. The type of crash seemed to be a better indicator of severe injuries than estimated speed at crash, and it could be considered to adapt general trauma triage protocols to the racetrack settings. Using the context of a study setting with medical professionals at the victim’s side within a few minutes after an accident offers unique possibilities to increase our understanding of clinical assessment in the very early state after an accident. Therefore, such study settings may benefit both sports medicine contexts and general trauma care.

## Data Availability

The datasets generated during and analysed during the current study are available from the corresponding author on reasonable request.

## Abbreviations

BF: Breathing frequency
CI: Confidence interval
GCS: Glasgow Coma Scale
HR: Heart rate
ISS: Injury severity score
NISS: New injury severity score
SpO2: Peripheral capillary oxygen saturation
SD: Standard deviations
SweTrau: Swedish Trauma Registry
SBT: Systolic blood pressure
